# Is There a “Gestalt Bias” in Indulgence? Subjectively Constructing Food Units Into Wholes (vs. Parts) Increases Desire to Eat and Actual Consumption

**DOI:** 10.3389/fpsyg.2021.671299

**Published:** 2021-06-15

**Authors:** Yannick Joye, Sabrina Bruyneel, Bob M. Fennis

**Affiliations:** ^1^ISM University of Management and Economics, Vilnius, Lithuania; ^2^Center of Expertise in Economics, Vilnius University, Vilnius, Lithuania; ^3^Behavioral Engineering Research Group, KU Leuven, Leuven, Belgium; ^4^Department of Marketing, University of Groningen, Groningen, Netherlands

**Keywords:** unit bias effect, portion size effect, perceptual grouping, Gestalt psychology, consumption, desire to eat

## Abstract

In the present work we extend research into the unit bias effect and its extension—the portion size effect—by demonstrating the existence of a “Gestalt bias.” Drawing on the tenets of Gestalt psychology, we show that a unit bias effect can be observed for food portions that are composed of identical basic units, but which are subjectively grouped into, or perceived as a Gestalt—a larger whole. In three studies, we find that such subjectively constructed food wholes constitute a new (perceptual) unit that is perceived bigger than the units it is constructed from, thereby prompting increased eating and desire to eat.

## Introduction

In times when obesity takes on almost epidemic proportions, researchers are increasingly looking at how manipulating the presentation and size of food servings can nudge people into healthier eating habits. Guided by this, scholars have discovered that eating behavior/decisions can be influenced by the “unit bias” (Geier et al., 2006)—the tendency for people to perceive any given unit of food as the default, regardless of its actual volume or physical attributes and thus as the appropriate amount to eat—or by its extension, the “portion size effect” (Rolls et al., 2006)—the tendency for people to perceive any portion size as the default and thus to consume more of an objectively larger than smaller portion. Indeed, Geier et al. (2006) define the unit bias (presumably underlying the portion size effect) as the tendency to “think that a unit of some entity (with certain constraints) is the appropriate and optimal amount” (p. 521). In their studies, participants tended to choose and wanted to consume a greater amount of Tootsie Rolls and pretzels when these were offered in a large as opposed to a smaller sized unit (Geier et al., 2006).

Importantly, the common methodological paradigm used in studies on the unit bias and portion size effect is to manipulate units/portions by creating objective volume, weight, or size differences between food units/portions, showing that a higher volume, weight or size induces increased food desire and/or consumption [see also: Zlatevska et al. (2014), Kerameas et al. (2015), Vandenbroele et al. (2019)]. But are such objective volume, weight or size differences between food items a sufficient, or even necessary condition to nudge consumers into eating more, possibly even exceeding their energy needs? In this paper, we argue that they are not, but instead propose that these effects are a particular instance of the more generic phenomenon that *perception drives consumption*—the tendency for appetitive motivation and intake to be a function of a *subjective construal* of volume, weight or size of food options rather than of objective differences between food options.

Note that in the unit bias and portion size paradigm, subjectively construed differences between food options are typically conflated with actual, objective differences (e.g., Geier et al., 2006; Kerameas et al., 2015; Vandenbroele et al., 2019). In the present work, we therefore aim to disentangle the perceptual differences between food units from their “objective” quantity differences and argue that such perceptual differences suffice in affecting food desire and consumption. We will specifically show that consumers' appetitive motivation and food consumption are not necessarily driven by objective quantity differences between food portions and units, but rather by their subjective construal and ensuing perceptual differences between portions and units, even when objective quantity differences between them are kept constant.

### A Gestalt Bias in Food Choice

In the present paper, we aim to extend the literature on the unit bias and portion size effects by demonstrating the existence of an underlying perceptual driver of these phenomena, which we coin a “*Gestalt bias*” in food choice. This proposed bias builds on one of the classic cornerstones in perceptual psychology: Gestalt psychology, developed by Max Wertheimer, Wolfgang Köhler, and Kurt Koffka in the early twentieth century (Koffka, 1935; Wagemans et al., 2012). Gestalt psychologists claim that we do not perceive the world as a collection of individual perceptual components, but they contend that we construe the elements in our perceptual field into grouped entities based on the extent to which we perceive them to “hang together” to form larger perceptual units or Gestalts (Wagemans et al., 2012; Guberman, 2015).

Gestalt psychologists have proposed various principles that lead us to perceive, and hence group, perceptual elements into larger wholes, such as the principle of “proximity”—the tendency to perceptually group elements together to the extent that they are in close proximity—and the principle of “closure”—the tendency to perceptually close gaps between distinct elements (Wagemans et al., 2012; Guberman, 2015). In the present three studies we apply these principles to the food domain, with the purpose of showing that food desire and consumption increase when basic food units are perceptually grouped into a larger whole or Gestalt, compared to their non-grouped, yet quantitatively identical counterparts. In Study 1 and 2, we focus on the impact of the Gestalt principle of proximity, while in Study 3 we focus on the Gestalt principle of closure.

In the food presentations across our studies, we expect that a subjectively construed food whole constitutes a new higher-order perceptual unit, irreducible to the basic units it is composed from. Because the size/volume of that higher-order unit/whole is by definition larger than its individual constituent units, it follows from research on the unit bias and portion size effect that consumers will desire and consume more of food when those constituent units are psychologically grouped into a larger whole rather than left in parts. We expect this to happen despite the fact that the total amount of food is objectively *identical* across ungrouped and grouped arrangements.

### Qualifying the Gestalt Bias in Food Choice

Note that the impact of a Gestalt bias (but *not* necessarily of the unit bias and portion size bias) will hinge on the tendency to see “the forest for the trees” and hence to apply its grouping principles to create larger wholes from constituent parts—in the present research the principles of proximity and closure. While Gestalt psychologists propose that this tendency is a perceptual default (cf., Wagemans et al., 2012), it nevertheless may well be amenable to contextual or situational differences. If so, then converging evidence for our notions would be provided if the proposed impact of a Gestalt bias on consumption would be particularly pronounced when people are induced to use higher order as opposed to lower order perceptual grouping. We explicitly test this in Study 2 using the so-called Navon letter task (Navon, 1977).

The present research also aims to rule out the role of a possible confound and rival account. That is, using higher order perceptual organization may inadvertently also induce what is termed a higher “construal level” (McCrea et al., 2012). This higher order, more abstract mindset typically allows people to transcend the here and now and see “the bigger scheme of things,” not only perceptually, but also in terms of the semantic and temporal construal of objects and events, goals, preferences, and self-regulatory strategies (see e.g., Trope and Liberman, 2010; Wiebenga and Fennis, 2014; De Vries and Fennis, 2019). Almost ironically, such higher construal levels have been shown to be associated with enhanced self-control, a focus on long term goals, and a *decreased*, not increased, sensitivity to (food) temptations and indulgence [see Fujita et al. (2006), Price et al. (2016), MacGregor et al. (2017)]. Thus, to the extent that a tendency for using higher order perceptual Gestalts also induces a more abstract construal level, predictions based on unit bias and portion size research diverge from predictions based on construal level theory, with the former implying *more* and the latter implying *less* indulgence following exposure to higher order Gestalts. The third and last study was designed to explicitly pit these two frameworks against each other to assess which account would be favored by the data.

### The Present Research

We conducted three studies to test this Gestalt bias on consumer indulgence of palatable foods, both in the lab (Study 1, 2) and online (Study 3), while keeping total food volume and unit sizes constant. In these studies, we assessed the impact of the proposed Gestalt bias on two consumption indicators—actual consumption (Study 1) and desire to eat (Study 1, 2 and 3). While studies on the unit bias and portion size effect often rely on actual consumption (Zlatevska et al., 2014), the appetitive motivation of *desire to eat* has been shown to align closely with actual food intake [e.g., Cornell et al., 1989; Rogers and Hardman, 2015; see Boswell and Kober (2016) for a meta-analysis] and has even been used *in lieu of* actual intake in portion size effect research (e.g., Burger et al., 2011). These properties render desire to eat a suitable candidate to study our Gestalt bias in an online context (Study 3), without compromising internal or construct validity.

## Study 1: Marshmallows Presented Either in Wholes Or Parts

Study 1 was a first test of the Gestalt bias in food choice. In this study, the basic food units were marshmallows (*n* = 12, in both conditions), which were presented to participants in either of two ways, manipulating the Gestalt principle of proximity (i.e., the tendency to perceptually group elements together when they are close to one another). In the “parts” condition, the twelve marshmallows were loosely arranged on a plate such that the individual unit(s) were individual marshmallows, whereas in the “whole” condition the twelve individual marshmallows were presented as kebabs, such that the focal unit was a higher-order whole, derived from perceptually grouping individual marshmallows. Although in both conditions the food portion consisted of the same discrete food units, *perceived* size differences between the focal units [i.e., whole (kebabs) vs. individual marshmallows/parts] should nudge differential indulgence in terms of desire to eat and/or actual consumption.

### Participants and Design

For this lab experiment, we analyzed data of 80 university students^1^ who provided informed consent and were not on a diet (age: *M* = 19.61, *SD* = 2.27; 52 females). The study used a single-factor design, with food organization (whole vs. parts) as the between-subjects variable, and desire to eat and actual consumption as the dependent variables. For all studies we used convenience samples consisting of as many participants as data collection time and budget would allow. Nevertheless, to assess statistical power, we used G^*^Power (Faul et al., 2007) to perform sensitivity analyses for this and the next studies to assess the minimal effect size the studies were able to reliably pick up with 80% power, given their actual sample size, and α = 0.05. Our studies proved sensitive enough to pick up small to medium effect sizes that are typical of research in (applied) psychology [see Richard et al. (2003)]. For the present study (using MANOVA, global effects), this sensitivity analysis yielded a minimal effect size of *f* = 0.35.

### Procedure and Measures

After introducing participants to the study, asking for informed consent, and requesting demographics, the experimenter brought each participant a (disposable) plate with twelve marshmallows on it. The marshmallows were presented either on wooden skewers as kebabs on the plate (“whole” condition) or were loosely arranged on the plate (“parts” condition). Participants were randomly assigned to one of the two conditions.

In the first phase of the study, participants had to rate ten filler statements about the marshmallows, to ensure that they would pay attention to the arrangement of the marshmallows (e.g., “The marshmallows appear to be soft”; 7-point scale ranging from “1 = not at all” to “7 = very much”). Directly after this, we assessed their desire to eat using six eating-related items (e.g., “How tasty does this candy look to you?”; “How much would you like to taste this candy right now?”), using 7-point scales ranging from “1 = not (tasty) at all” to “7 = very much/tasty”. We created a desire to eat index by averaging the scores on the six items (*M* = 4.24, *SD* = 1.42; α = 0.91; see the Appendix for all items). Note that during this first phase of the study, participants were asked to refrain from eating any marshmallows.

In the second phase, we invited participants to taste the marshmallows. We encouraged free tasting without any additional requirements, but asked participants to evaluate ten filler statements about the candy (e.g., “The marshmallows taste sweet”), using a 7-point scale ranging from “1 = not at all” to “7 = very much,” to ensure that they would take enough time tasting. The number of marshmallows consumed served as our measure of actual food intake. The study ended with debriefing participants about the aims and structure of the study.

### Statistical Analyses

For the target analyses, we used MANOVA, given the presumed correlation between desire to eat and actual consumption, with these two indices as dependent variables and food organization (whole vs. parts) as independent variable. We followed up the MANOVA with univariate analyses of variance (ANOVA).

### Results and Discussion

Replicating earlier research (Boswell and Kober, 2016), we indeed observed a significant, medium to large correlation between desire to eat and actual consumption, *r*(80) = 0.56, *p* < 0.001. Because of this close association, we performed our MANOVA with food organization (whole vs. parts) as the between-subjects factor, and with desire to eat and actual consumption as dependent variables. This analysis showed an impact of food organization, although the result fell slightly short of conventional significance threshold levels, *F*_(2,77)_ = 2.83, *p* = 0.065, η^2^_*p*_ = 0.07. Univariate analyses of variance revealed that while food organization did not affect desire to eat [whole: *M* = 4.29, *SD* = 1.49; parts: *M* = 4.18, *SD* = 1.36; *F*_(1,78)_ = 0.12, *p* = 0.733, η^2^_*p*_ < 0.01], it did have a significant impact on actual consumption, *F*_(1,78)_ = 4.61, *p* = 0.035, η^2^_*p*_ = 0.06, with participants eating significantly more marshmallows in the whole (*M* = 3.84, *SD* = 2.53) than in the parts condition (*M* = 2.72, *SD* = 2.08)^2^.

The current study shows that participants ate more marshmallows when these were organized into a whole (i.e., kebabs) than when they were loosely arranged on a plate. This result is surprising because from an affordance perspective (e.g., Kaaronen, 2017) one would actually expect that the whole condition would nudge against indulgence, since participants had to exert some effort to pull marshmallows from the skewers. Despite finding the proposed effect on actual consumption, the effect on desire to eat was not statistically significant. This might be because the answers to the filler statements about the marshmallows may have created an anchor for responses to the subsequent desire to eat statements, thus canceling out any meaningful variance as a function of our independent variable.

Our results provide first support for a Gestalt bias in food choice and thus extend research on the unit bias and portion size effect by showing that these tendencies do not necessarily require an objective difference between the volume or size of (the basic food units in) food portions. Instead, these effects are also evinced when constituent food parts are identical, yet perceptually grouped into larger wholes. Despite these results, one could argue that they are due to a fairly liberal experimental manipulation, since offering marshmallows on skewers offers a strong cue to grouped organization and thus almost “imposes” the perception of a whole. In Study 2 we aimed to address this issue by examining how desire to eat would be affected by simply prompting participants to *focus* on the whole or parts of a given food arrangement, while keeping food organization, volume and size constant across conditions.

## Study 2: Focusing on the Whole Or Parts of Chocolate Navon Letters

In Study 2 we aimed to find converging evidence for the proposed Gestalt bias while at the same time ruling out potential (visual) confounds (e.g., presence/absence of symmetry, orderliness, use of skewers). Therefore, we did not manipulate the organization of food itself, but offered participants identical food organizations, and encouraged them to focus on either the food whole, or on the units/parts constituting the whole. We specifically manipulated focus by either (or not) mobilizing the principle of proximity, i.e., the tendency to perceptually group elements together when they are close to one another. We did so by prompting participants to focus on the global vs. local structure of identical “Navon letters” (letters composed from smaller, different letters; Navon, 1977), which—for the purpose of this study—were made out of chocolate (rather than print) letters.

By using an adapted version of the Navon letter task, this study also directly tested a fundamental assumption underlying the Gestalt bias (but not necessarily the unit or portion size bias)—the assumption that food desire and/or consumption would increase mainly to the extent that people indeed show a tendency to see “the (larger) forest for the (constituent) trees.” Thus, the proposed effects should be particularly pronounced when people are induced to use higher order as opposed to lower order perceptual grouping using the Navon task.

As it was practically unfeasible to let participants actually taste chocolate letters in the lab (given the large size of the individual letters, and the large volumes of chocolate that would be required), we probed participants' desire to eat the chocolate letters. We expected that desire to eat chocolate letters would be higher after having focused on the whole than on the individual parts/letters, because the higher-order Navon letters are by definition larger than the individual chocolate letters they are composed from. We additionally examined whether the Gestalt bias would remain limited to the Gestalt-inducing food (i.e., chocolate letters), or would also spill over to other, unrelated palatable food items.

### Participants and Design

For this lab experiment, we analyzed data of 129 university students who provided informed consent and were not on a diet (age: *M* = 20.57, *SD* = 2.45; 74 females). The study used a mixed design with food focus (whole vs. parts) as the between-subjects variable, type of food (chocolate letters vs. other sweets) as the within-subjects variable, and desire to eat as the dependent variable. For this study, a sensitivity analysis (using G^*^Power, for ANOVA, repeated measures, within/between interaction) yielded 80% power to detect a minimal effect size of *f* = 0.11, given α = 0.05, the sample size, and the mixed design of the study.

### Procedure and Measures

**Figure 1:**
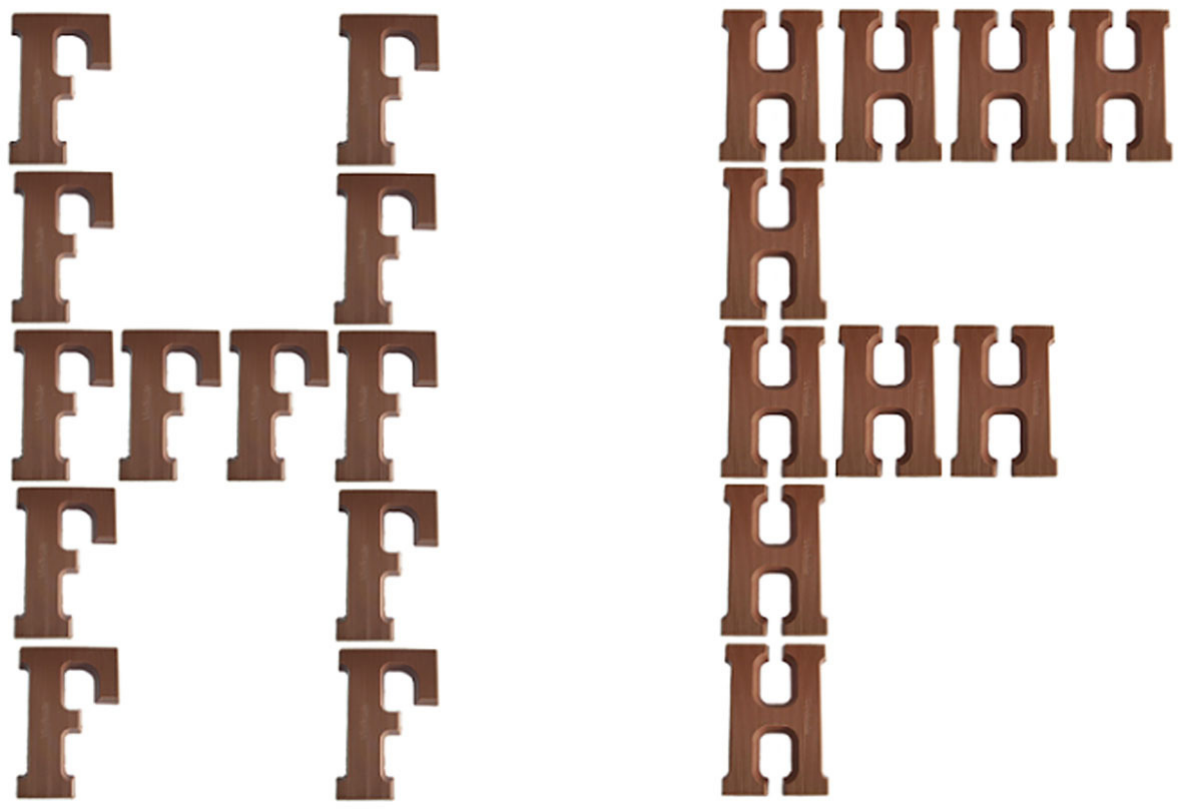
Sample pictures of “chocolate” Navon letters (Study 2). Left: sample stimulus used in the global condition; right: sample stimulus used in the local condition.

After introducing participants to the study, requesting informed consent and demographics, they were asked to carry out 128 trials of the Navon letter task. Each trial displayed a large letter that was made up of small photographs of chocolate letters (instead of print letters; Navon, 1977; Figure 1). Each individual Navon letter (390 by 482 pixels) was presented on a computer screen, and the vertical and horizontal parts of any given large letter consisted of a set of different smaller letters (e.g., a large F, consisting of smaller H's; Figure 1).

Participants were randomly assigned either to the global or local condition. In the global condition, they were shown 64 global H's (half of them composed of small F's, and the other half of small L's) and 64 global T's (half of them composed of small L's, and the other half of small F's). In the local condition, we showed participants 64 global L's (half of them consisting of small T's, and the other half of small H's) and 64 global F's (half of them composed of small T's, the other half of small H's). Presentation order of all Navon letters was randomized.

Each trial began with a fixation cross that was displayed in the middle of the screen for 500 ms. After this, a Navon letter appeared, and participants had to identify as quickly as possible whether the letter was a T or an H by pressing the corresponding button shown underneath the Navon letter (i.e., T to the left and H to the right). Because in the global vs. local condition T's and H's were, respectively, situated on the global vs. local level, the identification task prompted participants either to create a higher order food-related Gestalt or to refrain from doing so.

Immediately after the Navon letter task, participants were asked to watch and evaluate two types of sweets: the four chocolate letters we had used to create Navon letters (i.e., H, T, L, and F) and twelve other kinds of sweets (e.g., cake, candy). We assessed desire to eat using two eating-related measures (i.e., “How attractive does this … look to you right now?,” “How much would you like to eat from this … right now?”), using a 7-point scale ranging from “1 = not at all (attractive)” to “7 = very much/attractive.” We averaged scores to create a desire to eat index for the chocolate letters (*M* = 3.54, *SD* = 1.66; α = 0.98) and other sweets (*M* = 3.78, *SD* = 0.99; α = 0.91). Note that we chose for only two desire to eat items to avoid overtaxing participants (as they had to evaluate a total of sixteen sweets). The study ended with debriefing participants about the aim and structure of the study.

### Statistical Analyses

For the target analyses, and given our design, we analyzed the data using a 2 (focus) by 2 (type of sweets) mixed-design ANOVA with desire to eat as the main dependent variable. In addition, we followed up the mixed model ANOVA with univariate analyses of variance on desire to eat for each of the two types of sweets.

### Results and Discussion

**Figure 2:**
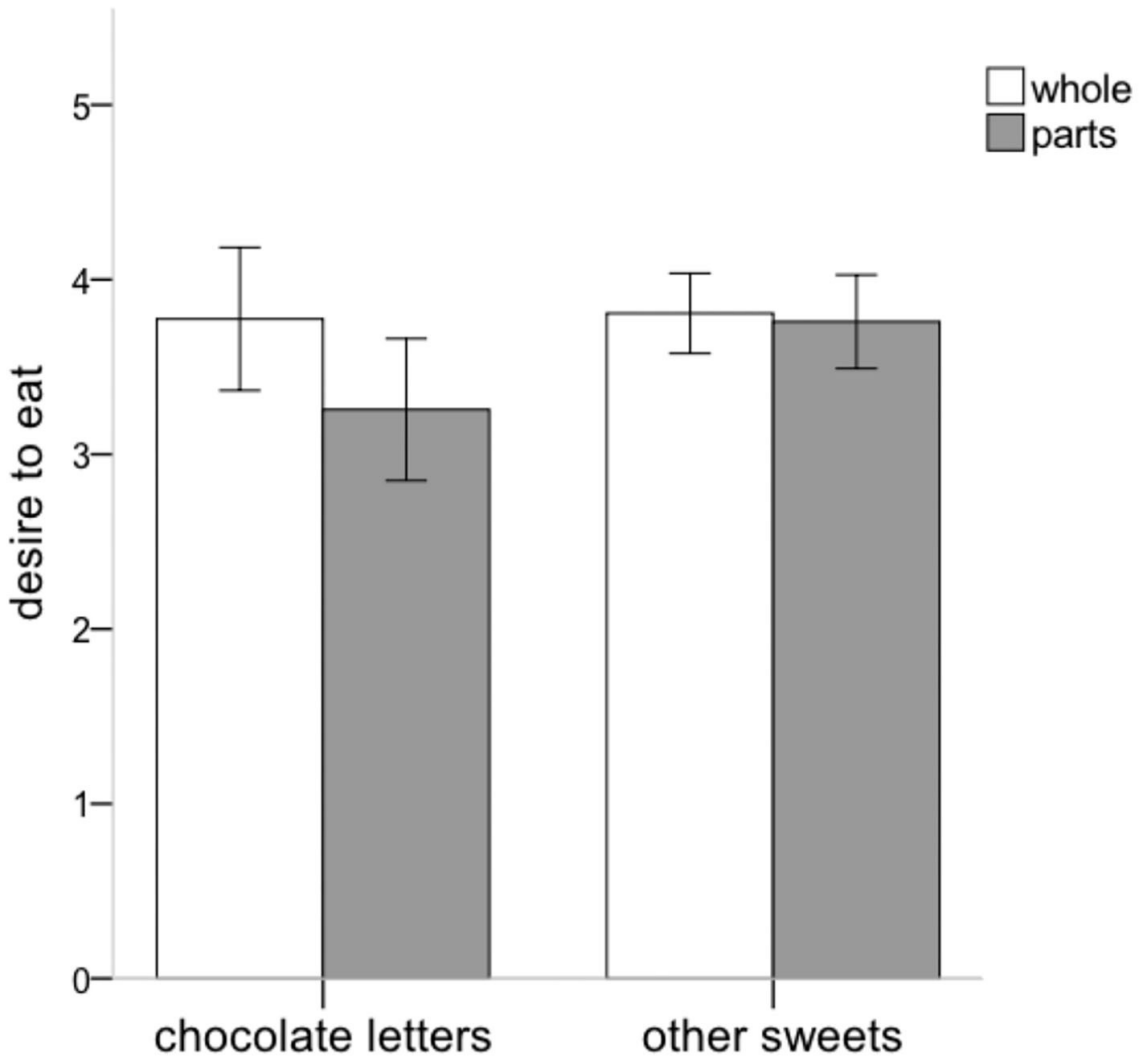
Graph of the focus by type of sweets interaction for desire to eat (Study 2).

The 2 (focus) by 2 (type of sweets) mixed-design ANOVA showed a main effect of type of sweets *F*_(1,127)_ = 5.29, *p* = 0.023, η^2^_*p*_ = 0.04, indicating that desire to eat was significantly higher for other sweets (*M* = 3.78*, SD* = 0.99) than for chocolate letters (*M* = 3.54, *SD* = 1.66). There was no significant main effect of focus, *F*_(1,127)_ = 1.81, *p* = 0.181, η^2^_*p*_ = 0.01 (global focus: *M* = 3.79, *SD* = 1.34; local focus: *M* = 3.51, *SD* = 1.29). Importantly, the analysis yielded an interaction effect between focus and type of sweets for desire to eat, *F*_(1,127)_ = 4.12, *p* = 0.045, η^2^_*p*_ = 0.03, indicating that the effect of focus differed for the two types of sweets (Figure 2).

Reflecting a Gestalt bias, the follow-up analyses showed that for the chocolate letters desire to eat tended to be higher in the global (*M* = 3.78, *SD* = 1.72) than in the local condition (*M* = 3.26, *SD* = 1.56), *F*_(1,127)_ = 3.18, *p* = 0.077, η^2^_*p*_ = 0.02. Desire to eat other sweets did not differ between both conditions, *F*_(1,127)_ = 0.07, *p* = 0.787, η^2^_*p*_ < 0.01 (global condition: *M* = 3.81, *SD* = 0.96; local condition: *M* = 3.76, *SD* = 1.03), indicating that the Gestalt bias remained limited to the type of food that was used for manipulating focus^3^.

In sum, using a controlled and well-established manipulation of perceptual focus, desire to eat chocolate letters was higher after focusing on the whole than on the parts of identical arrangements of chocolate (Navon) letters. The fact that the desire to eat chocolate letters in the whole condition is as high as the desire to eat other sweets (in both the whole and parts condition) might create the impression that focusing on the whole merely kept desire to eat unchanged, but lowered desire when focusing on the parts. This interpretation however hinges on the assumption that desire to eat is generally similar for chocolate letters and for the other sweets. Based on the available data, there is no way to ascertain this. Given that the other sweets presented to participants are presumably relatively popular and more familiar among the student public that participated in the study (more than chocolate letters), it appears more likely that the base-level desire to eat these types of sweets is simply higher than the desire for chocolate letters, while simultaneously being insensitive to the perceptual focus manipulation in the present study.

While the findings from the present study are consistent with a Gestalt bias, they are also in line with the global precedence effect, i.e., the (seemingly universal) tendency for human individuals to primarily and preferentially attend to the global (i.e., the whole) vs. local (i.e., the parts) properties of (visual) stimuli (Kimchi, 1992). Increased desire to eat food presented as a whole (vs. parts) may thus merely reflect this preference for the whole rather than a Gestalt bias. In the next study we aimed to rule out this alternative explanation by comparing food arrangements that were both perceived as wholes but that differed in perceived size. If desire to eat would be highest for the *largest* food whole (while keeping wholeness constant across conditions), then this would favor a Gestalt bias over a global precedence account.

## Study 3: Comparing Food Wholes Differing in Perceived Size

**Figure 3:**
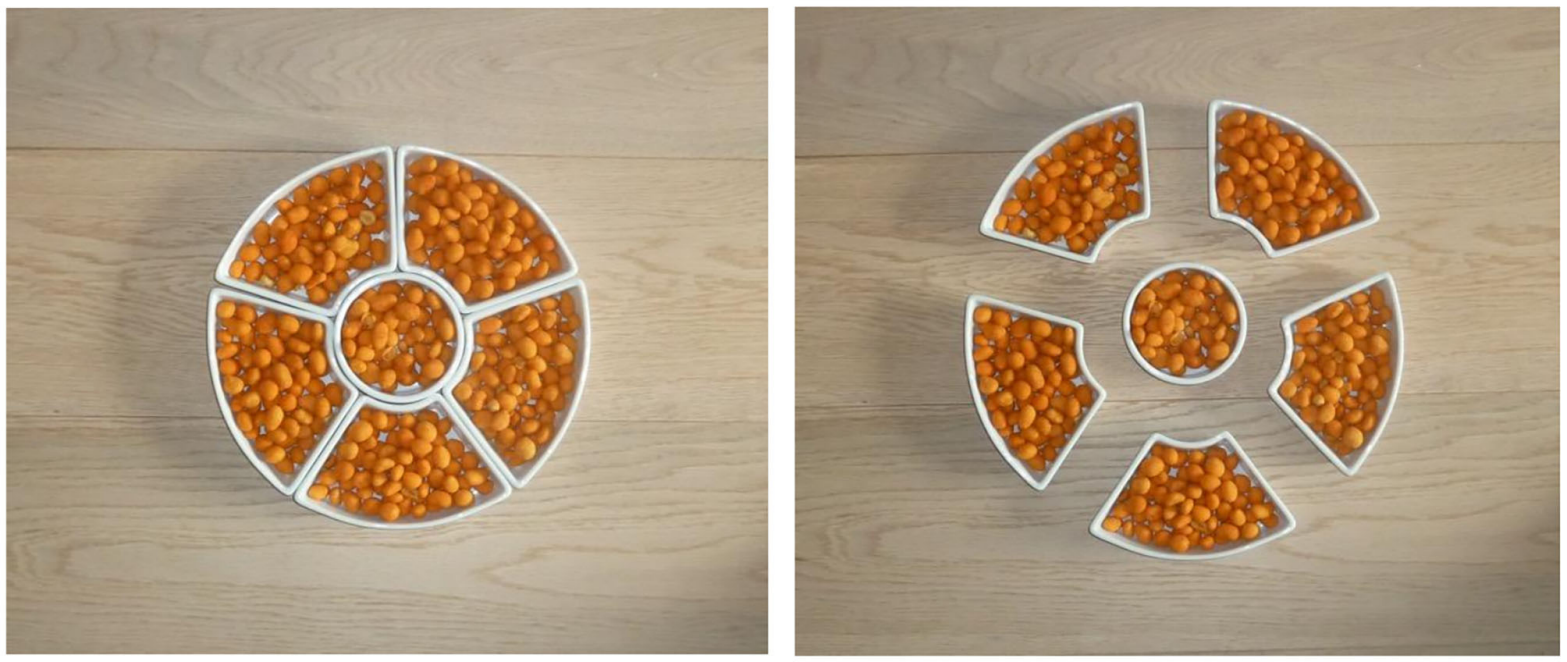
Photographs of the tight (left) and loose (right) arrangement of cups (Study 3).

In Study 3, we examined whether mere perceived size differences between similar food wholes would suffice to lead to differences in desire to eat, as the Gestalt bias suggests. We tested this notion by arranging lazy susan cups filled with nuts into a circular shape, either in a tight or in a looser arrangement (Figure 3). While in the looser arrangement, the individual cups were not joined together, we reasoned that participants would nevertheless perceive the collection of cups to form a circle, due to the Gestalt principle of closure (i.e., tendency to perceptually close gaps between distinct elements). As the circumference of the (imaginary) circle in the looser arrangement is by definition larger than the circumference of the tight arrangement of cups, we expected that—by the logic of the Gestalt bias—participants would have a higher desire to eat nuts in the former than the latter condition.

In addition to manipulating perceived food size, we also manipulated construal level (Fujita et al., 2006) to rule out this construct as a possible confound and rival account of our findings. As argued in the Introduction, a higher order perceptual organization may have inadvertently also induced a higher construal level in participants, i.e., a higher order, more abstract conceptual mindset. Higher construal levels have been associated with more self-control, a focus on long-term goals, and decreased sensitivity to (food) temptations and indulgence [see Fujita et al. (2006), MacGregor et al. (2017)]. Thus, our predictions regarding a Gestalt bias are opposite to those for a high construal level, with the former implying *more* and the latter implying *less* indulgence following exposure to higher order Gestalts. Orthogonally manipulating both the visual food organization as well as construal level allowed us to pit both accounts against each other and to assess the empirical support for both.

### Participants and Design

For this online (*Prolific*) experiment, we analyzed data of 287 participants who provided informed consent an who were not on a diet (age: *M* = 30.90, *SD* = 10.96; 141 females, 2 “neither male, nor female”). The study used a 2 (type of food arrangement: loose vs. tight) by 2 (construal level: high vs. low) between-subjects factorial design with desire to eat as the main dependent variable. For the present study, a sensitivity analysis (using G^*^Power, for ANOVA, fixed effects, special, main effects and interactions), yielded 80% power to detect a minimal effect size of *f* = 0.17, given α = 0.05, and the design and sample size of the study.

### Procedure and Measures

After requesting informed consent and demographical information, we randomly assigned participants to the two conditions. We first manipulated construal level using a validated task (Fujita et al., 2006) where we asked participants either to come up with twenty superordinate category labels of common objects (e.g., “a CAR is an example of…”), inducing a high level, abstract mindset, or with subordinate exemplars (e.g., “an example of a CAR is…”), inducing a low level, concrete mindset. Following this, we showed participants a photograph of six lazy susan cups filled with party nuts (636 by 540 pixels). In the tight condition, the six cups formed a tightly arranged whole, whereas in loose condition we activated the Gestalt principle of closure—the cups were somewhat pulled apart, but still close enough to be perceived as a (comparatively larger) whole (see Figure 3). Participants were asked to carefully inspect the target photograph, and then to click away to the next page of the survey.

Next, we measured participants' desire to eat the party nuts using 6 items (e.g., “These party nuts look very tasty,” “I get hungry from watching these party nuts,” 7-point scale ranging from “strongly disagree” to “strongly agree”; *M* = 4.08, *SD* = 1.44; α = 0.94; see Appendix for all items), and gauged how easy it was for them to visually process/perceive the lazy susan cups using the perceptual fluency scale by Graf et al. (2018; see Appendix for all items; *M* = 5.44, *SD* = 1.26; α = 0.82). We then asked participants if the arrangement of cups containing the nuts looked like a whole (7-point scale ranging from “strongly disagree” to “strongly agree”), and—to obtain a proxy for the size of the food arrangement—requested them to estimate the total amount of nuts (grams) across all six cups (sliding scale ranging from 0 to 500 g). During all evaluations, a smaller copy (300 by 254 pixels) of the target picture was visible to participants. The study ended with debriefing participants about the aim and structure of the study.

### Statistical Analyses

For the preliminary analyses assessing the success of the manipulations, confounding effects, and alternate accounts, we used 2 (type of food arrangement) by 2 (construal level) ANOVAs with the respective constructs as dependent variables (see above). For the main analyses we similarly used 2 (type of food arrangement) by 2 (construal level) ANOVAs with portion estimates (in grams) and desire to eat as the dependent variables.

### Results and Discussion

#### Preliminary Analyses

The 2 (type of food arrangement) by 2 (construal level) ANOVA, revealed that participants did not detect any difference in wholeness between the tight (*M* = 5.05, *SD* = 1.56) and loose (*M* = 5.01, *SD* = 1.68) food arrangements, *F*_(1,283)_ = 0.02, *p* = 0.876, η^2^_*p*_ < 0.01. This result suggests that the loose arrangement was still grouped as a perceptual whole, which is consistent with the assumption that the principle of closure governed the perception of the loose arrangement. A second two-way ANOVA showed that participants experienced the tight arrangement of cups as easier to process (*M* = 5.60, *SD* = 1.22) than its loose counterpart (*M* = 5.29, *SD* = 1.29), *F*_(1,283)_ = 4.45, *p* = 0.036, η^2^_*p*_ = 0.02, which possibly reflects participants' higher familiarity with tight vs. loose arrangements of cups.

Both ANOVAs yielded no significant main effect of construal level on wholeness, *F*_(1,283)_ = 0.01, *p* = 0.912, η^2^_*p*_ < 0.01 (low construal: *M* = 5.02, *SD* = 1.58; high construal: *M* = 5.04, *SD* = 1.66) or on fluency, *F*_(1,283)_ = 0.45, *p* = 0.502, η^2^_*p*_ < 0.01 (low construal: *M* = 5.48, *SD* = 1.25; high construal: *M* = 5.40, *SD* = 1.27). Moreover, there were no significant interaction effects between type of food arrangement and construal level on both measures [wholeness: *F*_(1,283)_ = 0.46, *p* = 0.501, η^2^_*p*_ < 0.01; fluency: *F*_(1,283)_ = 0.23, *p* = 0.636, η^2^_*p*_ < 0.01]. These findings align with our expectation that both the loose and tight arrangements of cups were perceived as having similar levels of wholeness, thus ruling out global precedence as an alternate account for any effects found.

#### Main Analyses

The 2 (type of food arrangement) by 2 (construal level) ANOVA on the portion estimate (in grams), indicated that participants estimated that in the loose arrangement, the cups contained more grams of nuts (*M* = 256.96, *SD* = 101.28) than in the tight arrangement (*M* = 226.95, *SD* = 92.89) *F*_(1,282)_ = 6.64, *p* = 0.010, η^2^_*p*_ = 0.02^4^. This is in line with our assumption that the looser arrangement is indeed perceived as more voluminous than the tighter one, even though both are objectively identical in volume. Additionally, and in line with the Gestalt bias, participants showed a greater desire to eat in the loose (*M* = 4.32, *SD* = 1.34) than in the tight condition (*M* = 3.82, *SD* = 1.50), *F*_(1,283)_ = 9.02, *p* = 0.003, η^2^_*p*_ = 0.03^5^.

Note that both ANOVAs revealed no significant main effect of construal level on the portion estimate, *F*_(1,282)_ = 0.57, *p* = 0.449, η^2^_*p*_ < 0.01 (low construal: *M* = 238.24, *SD* = 97.87; high construal: *M* = 246.32, *SD* = 98.87), or on desire to eat, *F*_(1,283)_ = 0.01, *p* = 0.938, η^2^_*p*_ < 0.01 (low construal: *M* = 4.09, *SD* = 1.44; high construal: *M* = 4.06, *SD* = 1.44). There was furthermore no significant interaction between type of food arrangement and construal level [portion estimate: *F*_(1,282)_ = 0.99, *p* = 0.321, η^2^_*p*_ < 0.01; desire to eat: *F*_(1,283)_ = 0.01, *p* = 0.936, η^2^_*p*_ < 0.01].

In agreement with the Gestalt bias, and independent of construal level, participants thus showed a greater desire to eat from the largest psychologically perceived food arrangement. The present results rule out that the Gestalt bias is confounded with high construal level, which would have assumed an interaction effect, something that the present results do not support.

## General Discussion

With the current paper we extend previous work on the unit bias and portion size effect by proposing and testing a Gestalt bias. We proposed and tested the notion that perception drives appetite, rather than objective size differences in food options. Across three studies, participants experienced a greater desire to eat (Study 2, 3), and consumed more of palatable foods (Study 1) when basic food units were perceptually grouped into larger wholes than when those same units were left in parts (Study 1, 2), or when they constituted a smaller whole (Study 3). As such, the present findings highlight the role of a driver of the unit bias and portion size effect that has hitherto remained hidden or implicit in empirical research (Zlatevska et al., 2014). That is, work examining these biases has predominantly relied on food manipulation paradigms where food options not only differed perceptually, but also objectively, in terms of size, volume, and/or weight. By pulling these two dimensions apart and keeping objective dimensions constant, while merely varying perceptual properties of food options, we demonstrated that perceptual (i.e., subjective) differences suffice for observing an impact on desire and/or actual food intake. Our findings thus extend the playing field of the unit bias and portion size effect to those instances where objective differences between food options are absent, but perceptual differences (in terms of perceptual grouping) are made salient.

### Limitations and Future Research Directions

In the present work we limited our research to examining the Gestalt principles of proximity and closure in the food domain. Future research might extend the present findings by focusing on the role of as yet untested Gestalt principles in affecting eating desire and/or actual consumption, such as the principles of similarity (i.e., the extent to which food options share perceptual features), good continuation (i.e., the extent to which a subsequent food option is seen as a logical perceptual continuation of its predecessor), or symmetry [i.e., the extent to which two food options mirror each other in terms of perceptual features, see Guberman (2015)].

Of course, research might also address some of the limitations of the present studies. For example, in Study 1, we observed an effect of the Gestalt bias on actual food intake, but not on food desire. Apart from the possible anchoring role of the filler questions used in that study, another explanation may be the use of different measures of eating desire across our studies. While this heterogeneity of measures was intentional to provide more robust evidence for our notions, future research might additionally seek to replicate the present findings using a fixed set of pre-validated, calibrated measures assessing food craving, and/or positive eating experiences (see e.g., White et al., 2002; Sproesser et al., 2018). Since only one study (Study 1) involved actual food intake, more research is also needed to further support the role of the Gestalt bias in directly affecting actual consumption. Moreover, to salvage some of the additional inherent limitations of our set of studies, future research might also assess the robustness of the present findings among more heterogeneous samples, across lab, online and field settings, and for different types of (more or less) hedonic foods.

An assumption underlying the Gestalt bias is that it operates as a perceptual default (cf., Ashby et al., 2003). This seems to imply that applying Gestalt principles would largely occur outside conscious awareness. However, to our knowledge, this assumption has not yet been systematically tested, and future research might take up this challenge. If indeed so, then first of all, administering manipulation checks in future studies might be a challenge, since people cannot be expected to consciously access and express perceptions that occur non-consciously. Moreover, if this bias indeed occurs outside of awareness, we would expect its impact on food desire and choice to be more pronounced under conditions conducive of automaticity and non-consciousness, for example when consumers are distracted. This would constitute another line of evidence for the frequently recorded observation that people tend to consume more while distracted, for example when they are in the company of others while eating [see Ogden et al. (2013)]. Conversely, if it is indeed demonstrated that the Gestalt bias operates outside conscious awareness then a relatively recent food trend such as “mindful eating” [see Warren et al. (2017)] might well be less amenable to this bias.

Research might also focus on other implications of the present findings. For example, we found that the Gestalt bias is more pronounced when people are prompted to focus on larger wholes than on smaller parts (Study 2). This raises the possibility that individual differences in the tendency for perceiving and thinking in terms of larger categories would modulate the Gestalt bias. One candidate to further explore in this context might be individual differences in working memory capacity (Lewandowsky, 2011), while another may be the need for cognitive closure (Webster and Kruglanski, 1994).

### Implications for Health Promotion and Interventions

The present findings might also be the starting point of easy-to-implement interventions that can counter the undesirable effects of the Gestalt bias and its specific manifestations (e.g., portion size effect and unit bias). Perhaps one of the more obvious ways to counter the power of the Gestalt bias is by limiting the opportunity for perceptual grouping. The consumption of palatable foods such as cake or pizza should for example be reduced when the individual pieces do no longer constitute a rounded shape, but are presented in a mixed, more haphazard arrangement, so as to no longer constitute a larger perceptual whole.

The presumed automaticity that underlies the Gestalt bias can also pave the way for additional interventions aimed at attenuating its potentially adverse impact on indulgence. For example, Gestalt principles may well function as simple decision heuristics that shape consumers' consumption choices. Work on heuristic decision making (e.g., Fransen and Fennis, 2014) suggests that simply warning consumers of their tendency to mindlessly apply these perceptual grouping principles (either explicitly or implicitly) may suffice to reduce the tendency for a Gestalt bias in food choice. Moreover, raising such awareness may also prove to be effective in combating the Gestalt bias as it will directly interfere with the presumed automaticity of the process, and so may thwart its impact on food choice.

### Conclusion

In sum, our findings lend support to the notion of a Gestalt bias in food indulgence, while excluding alternative explanations, such as global precedence or construal level. Future research could go beyond our first test of this notion, not only by addressing some of its limitations (e.g., diversity of our measures), but also by looking into possible boundary conditions of the effect (e.g., does the effect also play for healthy foods?).

With the present research we add a tool to the repertoire of measures to battle the obesity epidemic. The existence of a Gestalt bias shows that food consumption/desire can be influenced when food is arranged in such a way that it is perceived as a higher-order (perceptual) unit, deemed bigger than the units it is constructed from. Conversely, the present findings suggest that consumption of palatable foods can be mitigated by simply shifting attention to the smaller details of a food serving, such as focusing on pizza slices rather than on the entire pizza.

In conclusion, in addition to extending research and theory regarding the unit bias and portion size effect, the current work also highlights how one of the oldest paradigms in perceptual psychology—Gestalt psychology—remains relevant for understanding and steering present-day consumer behavior and decisions.

## Data Availability Statement

The raw data supporting the conclusions of this article will be made available by the authors, without undue reservation.

## Ethics Statement

Ethical review and approval was not required for the study on human participants in accordance with the local legislation and institutional requirements. The patients/participants provided their written informed consent to participate in this study.

## Author Contributions

YJ: conceptualization, methodology, formal analysis, and writing. SB and BF: conceptualization, methodology, and writing. All authors contributed to the article and approved the submitted version.

## Conflict of Interest

The authors declare that the research was conducted in the absence of any commercial or financial relationships that could be construed as a potential conflict of interest.

## Footnotes

^1^One additional participant, who was not on a diet, completed the study, but due to a coding error the condition she was in was not recorded.

^2^Reanalyzing the data while including gender as a covariate yielded the following results: effect of food organization (whole vs. parts) on desire to eat, *F*_(1,77)_ = 0.10, *p* = 0.756, η^2^_*p*_ < 0.01, and on actual consumption, *F*_(1,77)_ = 4.35, *p* = 0.040, η^2^_*p*_ = 0.05.

^3^Reanalyzing the data while including gender as a covariate yielded the following results: effect of global (vs. local) focus on desire to eat chocolate letters, *F*_(1,126)_ = 3.08, *p* = 0.082, η^2^_*p*_ = 0.02, and other sweets, *F*_(1,126)_ = 0.07, *p* = 0.796, η^2^_*p*_ < 0.01.

^4^One participant did not submit an amount estimate.

^5^Reanalyzing the data while including gender as a covariate yielded the following results: effect of the loose (vs. tight) food arrangement on the desire to eat, *F*_(1,282)_ = 9.15, *p* = 0.003, η^2^_*p*_ = 0.03.

## Appendix

### Desire to Eat Items Used in Study 1

“How attractive does this candy look to you?,” “How much does watching this candy make you feel hungry?,” “How tasty does this candy look to you?,” “How much would you like to taste this candy right now?,” “How quickly are you planning to eat this type of candy again in the future?,” “How many extra minutes would you be willing to stay in the lab to taste the candy?”; all items scored either from “1 = not at all (attractive/hungry/tasty/quickly)” to “7 = very much (attractive/hungry/tasty/quickly)” or from “1” to “7 min.”

### Perceptual Fluency Items Used in Study 3 (Taken From Graf et al., 2018)

Seven-point bipolar scales with the following items: “difficult—easy”; “unclear—clear”; “disfluent—fluent”; “effortful—effortless”; “incomprehensible—comprehensible”

### Desire to Eat Items Used in Study 3

“These party nuts look very tasty,” “I get hungry from watching these party nuts,” “I would like to taste these party nuts right now,” “These party nuts look appealing,” “I soon would like to buy party nuts like these,” “I would enjoy eating these party nuts”; 7-point Likert scale ranging from “strongly disagree” to “strongly agree.”
